# The estrogen-injected female mouse: new insight into the etiology of PCOS

**DOI:** 10.1186/1477-7827-7-47

**Published:** 2009-05-18

**Authors:** John C Chapman, Soo Hong Min, Steven M Freeh, Sandra D Michael

**Affiliations:** 1 Department of Biological Sciences, Binghamton University, Binghamton, New York 13902-6000, USA; 2 Schering-Plough Research Institute, Kenilworth, New Jersey 07033-1300, USA

## Abstract

**Background:**

Female mice and rats injected with estrogen perinatally become anovulatory and develop follicular cysts. The current consensus is that this adverse response to estrogen involves the hypothalamus and occurs because of an estrogen-induced alteration in the GnRH delivery system. Whether or not this is true has yet to be firmly established. The present study examined an alternate possibility in which anovulation and cyst development occurs through an estrogen-induced disruption in the immune system, achieved through the intermediation of the thymus gland.

**Methods, Results and Conclusion:**

A putative role for the thymus in estrogen-induced anovulation and follicular cyst formation (a model of PCOS) was examined in female mice by removing the gland prior to estrogen injection. Whereas all intact, female mice injected with 20 ug estrogen at 5–7 days of age had ovaries with follicular cysts, no cysts were observed in animals in which thymectomy at 3 days of age preceded estrogen injection. In fact, after restoring immune function by thymocyte replacement, the majority of thymectomized, estrogen-injected mice had ovaries with corpora lutea. Thus, when estrogen is unable to act on the thymus, ovulation occurs and follicular cysts do not develop. This implicates the thymus in the cysts' genesis and discounts the role of the hypothalamus. Subsequent research established that the disease is transferable by lymphocyte infusion. Transfer took place between 100-day-old estrogen-injected and 15-day-old naïve mice only when recipients were thymectomized at 3 days of age. Thus, a prerequisite for cyst formation is the absence of regulatory T cells. Their absence in donor mice was judged to be the result of an estrogen-induced increase in the thymus' vascular permeability, causing de facto circumvention of the final stages of regulatory T cell development. The human thymus has a similar vulnerability to steroid action during the fetal stage. We propose that in utero exposure to excessive levels of steroids such as estrogen has a long-term effect on the ability of the thymus to produce regulatory T cells. In female offspring this can lead to PCOS.

## Background

Polycystic ovarian syndrome (PCOS) occurs in 5%–10% of all women of reproductive age [1,2]. The disease begins at menarche, and symptoms generally include oligomenhorrhea, amenorrhea, anovulation, cystic ovaries, an elevated LH/FSH ratio, obesity, hirsutism, and insulin resistance. Cystic ovaries produce high levels of androstenedione, testosterone, and 17αOH-progesterone. The cysts themselves are remnants of atretic follicles, fluid filled and devoid of granulosa cells. As reported in the scientific literature in 1935 [3] the etiology of PCOS still remains obscure. Three major hypotheses are [4]: 1) PCOS is due to a primary neuroendocrine defect leading to an exaggerated LH pulse frequency and amplitude; 2) the disease is caused by a deficiency in insulin action leading to hyperinsulinemia; and 3) the primary fault occurs in the ovary and involves changes in FSH response.

Basic symptoms of PCOS such as anovulation and follicular cysts are produced in female mice by injecting them with estrogen, testosterone, or cortisone prior to 10-days of age [5,6]. Significantly, during this same 10-day period the thymus gland is in its final stages of development. Interference with this process alters the evolution of 'self' versus 'nonself' recognition [7]. For example, thymectomy at 3-days of age prevents the production of regulatory T cells, and a number of autoimmune diseases ensue [8,9]. Evidence presented herein suggests that steroids also forestall the production of regulatory T cells. The resultant autoimmune disease in this instance is PCOS.

## Methods

### Animals and reagents

Female (C57Bl/6J × A/J)F_1 _(B6A) mice were used in the study. Parental stocks were purchased from Jackson Laboratory, Bar Harbor, ME. All mice were maintained in our animal care facility and cared for in accordance with institutional guidelines. Sesame oil, steroid hormones, Hanks balanced salt solution (HBSS), and Trypan Blue were purchased from Sigma Chemical Company; St. Louis, Missouri, USA. Lympholyte M was purchased from Cedarlane Laboratories, Ontario, Canada. All other reagents were purchased from Fisher Scientific, Hampton, NH.

### Treatments

#### Steroid injection

Neonatal B6A female mice were injected subcutaneously (sc) with 0.010 ml sesame oil:ethanol (9:1; v:v) (vehicle), or vehicle containing 20 μg of either estradiol-17β, testosterone, cortisol, or progesterone; or vehicle containing 10 μg diethlystilbestrol (DES).

#### Thymocyte infusion

Thymocytes were prepared from thymuses taken from mice killed by etherization. After weighing the thymus it was pressed between two glass slides and connective tissue teased away and discarded. The thymocytes were then suspended in 4 ml HBSS and centrifuged at 1000 × g for 10 min at 22°C. Following a second wash and recentrifugation, the cells were resuspended in 4 ml of HBSS and counted with a hemocytometer. The thymocyte suspension was then recentrifuged and the pellet resuspended in HBSS. Total volume of the suspension was adjusted to allow for an infusion of 20 million thymocytes per 0.10 ml HBSS. The infusions were administered to 15-day-old Tx-3 pups via a 1 ml plastic syringe outfitted with a 0.5 inch, 27 gauge needle inserted just above the "navel". The infusion site was subsequently covered with skin-bond adhesive. Immature female thymocyte donors were 7 days of age, and mature female thymocyte donors ranged from 60 days to 120 days of age.

#### Lymphocyte infusion

Splenocytes were isolated from spleens removed from decapitated female donors. Each spleen was weighed and placed on a stainless steel wire mesh (0.65 mm × 0.65 mm) suspended over a glass beaker. Using the rubber end of a 5 ml plastic syringe plunger, the spleen was mashed and the sheath discarded. The resultant splenocytes were washed from the mesh, and centrifuged at 250 × g for 10 min at 22°C. Subsequent treatments depended on the individual study. When splenocytes were used for counting, the pellet was resuspended in 3 ml HBSS; however, when prepared for infusion, the splenocyte pellet was resuspended in 12 ml HBSS. The suspension was then apportioned between two 15 ml conical plastic test tubes. Two ml of Lympholyte M were added to the bottom of each tube, and lymphocytes separated from erythrocytes by centrifugation at 1200 × g for 20 min at 22°C. Lymphocytes were recovered and suspended in 6 ml of HBSS and centrifuged at 250 × g for 10 min at 22°C. The lymphocyte pellet was resuspended in 3 ml of HBSS and counted with a hemocytometer. The suspension was recentrifuged and the pellet resuspended in HBSS. The total volume was adjusted to allow for an infusion of 20 million lymphocytes per 0.10 ml HBSS. Lymphocyte infusions were administered to 15-day-old Tx-3 pups as described for thymocyte infusions. Estrogen-injected female donors ranged from 100 days to 110 days of age.

### Cell counting

Cells were counted using the Trypan Blue Exclusion Test. For this procedure, 0.010 ml was removed from the 3 ml (splenocyte) and 4 ml (thymocyte) stock suspensions and added to a microcentrifuge tube containing 0.080 ml of HBSS and 0.010 ml of Trypan Blue dye. Viable cells were enumerated in a hemocytometer with the aid of a light microscope. Two quadrants were averaged for each sample. In general, there were few cells that did not exclude Trypan Blue, indicating the efficiency of the procedure in producing viable cells.

### Sacrifice, sample collection, and histology

After light etherization, the mice were killed by decapitation. Unless otherwise stated, all experimental animals were killed between 100 and 110 days of age. Ovaries were collected, placed in Bouins fixative for 24 hr, and then stored in 70% ethanol. Fixed ovaries were cleaned of adherent fat, weighed, and embedded in paraffin. Afterwards, they were sectioned at 5 μm, placed on a glass slide, and stained with Harris's hematoxylin and eosin Y. Stained sections were examined via light microscopy for the presence of follicular cysts, corpora lutea (CLs), and for ovarian dysgenesis. Ovaries were classified according to state of reproductive ability. For example, ovaries that contained follicles and CLs were considered to be from fertile mice. Ovaries that lacked CLs, and contained follicular cysts were considered to be from infertile mice. Ovaries that lacked CLs and follicles were deemed to be dysgenic.

### Other procedures

Thymectomy was performed on day 3 (Tx-3) by aspiration, as previously described [10]. Statistical analyses were performed on all experiments having multiple replicates, using ANOVA and the Student t test. All data are reported as mean ± standard error of the mean (S.E.M.).

## Results and discussion

Our first study (Table 1) was undertaken to determine if removing the thymus prior to estrogen injection could prevent anovulation and follicular cysts. Mice were thymectomized on day 3 (Tx-3) [10], and at 5–7 days of age given daily injections of estradiol-17β (E_2_) (Treatment 7); the thymus was essentially replaced after steroid treatment by infusing the Tx-3 animals on day 15 with 20 × 10^6 ^thymocytes from adult female donors [11]. Whereas all intact, 100-day-old female mice injected with E_2 _at 5–7 days of age (Treatment 2) had ovaries containing follicular cysts, none were observed in 100-day-old Treatment 7 animals in which thymectomy preceded E_2 _injection. In fact, the majority had ovaries with corpora lutea (CLs). Thus, when E_2 _is unable to act on the thymus, ovulation occurs and follicular cysts do not develop. This implicates the thymus in the cysts' genesis. Note that infusion of thymocytes from 7-day-old females neither prevented cysts (Treatment 5), nor restored immunocompetency to Tx-3 mice (Treatment 4). The reason for this will be discussed later.

**Table 1:** The prevention of follicular cysts in estrogen-injected (C57BL/6J × A/J) F_1 _(B6A) female mice by thymectomy and thymocyte replacement.

	Condition of Ovaries			
Treatment	CLs	Dysgenic	Cysts	Other
1. Sesame oil Days 5–7 [8]^a^	100%^b^ (14.7 ± 1.4)^c^	------	------	------
2. E2 Days 5–7 [17]	------	------	100% (10.8 ± 0.3)	------
3. Tx-3 [19]	47% (11.8 ± 0.6)	53% (2.6 ± 0.6)	------	------
4. Tx-3 + thymocytes from 7 day-old females [8]	13% (5.8)	------	63% (5.5 ± 0.8)	24% (3.9 ± 0.8)^d^
5. Tx-3 + E2 Days 5–7 + thymocytes from 7-day-old females [7]	14% (1.9)	43% (1.8 ± 0.8)	14% (5.9)	29% (2.0 ± 1.0)^e^
6. Tx-3 + thymocytes from adult females [18]	100% (9.9 ± 0.6)	------	------	------
7. Tx-3 + E2 Days 5–7 + thymocytes from adult females [11]	73% (12.4 ± 1.8)	------	------	27% (7.8 ± 0.3)^f^

Mice in treatments **(1) **and **(2) **were injected sc at 5–7 days of age with either sesame oil: ethanol (9:1; v:v) {vehicle}, or vehicle containing 20 μg estradiol-17β (E_2_). In treatment **(3)**, the females were thymectomized at 3 days of age (Tx-3). Treatments **(4) **and **(6) **consisted of infusing 15-day-old Tx-3 mice with 20 million thymocytes, isolated from thymuses of either 7-day-old or adult female B6A donors. In treatments **(5) **and **(7)**, Tx-3 females were injected with E_2 _at 5–7 days of age, and then infused with 20 million thymocytes, isolated from thymuses of either 7-day-old or adult female B6A donors. All recipient females were killed at 100 days of age and ovaries removed and examined histologically for corpora lutea (CLs), follicular cysts, and dysgenesis. Normal ovaries contained normal follicle populations and CLs. Dysgenic ovaries lacked all follicle populations. Cystic ovaries contained follicular cysts, and lacked CLs.

^a^Number of animals in each group.

^b^Percentage of animals with similar ovarian histology.

^c^Average weight of paired fixed ovaries (mg ± S.E.M.).

^d^Ovaries with cysts and CLs.

^e^Dysgenic ovaries with cysts.

^f^Ovaries with normal follicle populations, but lacking both CLs and follicular cysts.

**Figure 1:**
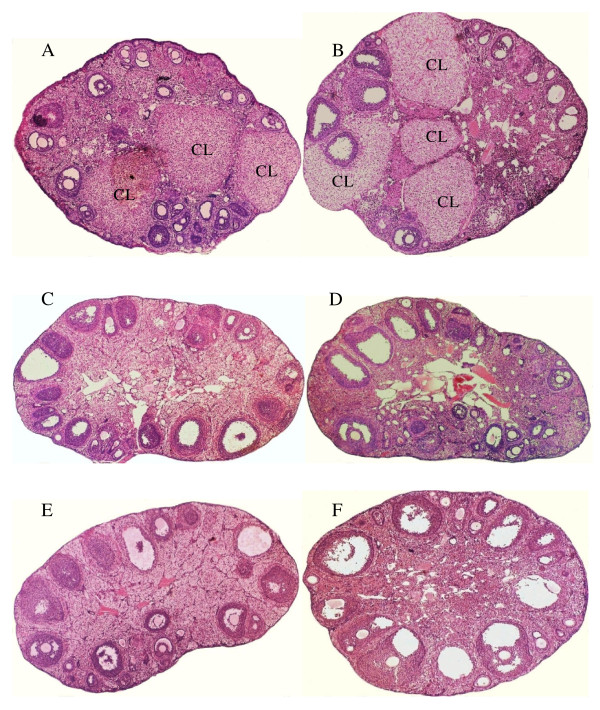
**Creation of follicular cysts in Tx-3 female mice by the infusion of lymphocytes from E_2_- and DES-injected female B6A mice**. Cross-section of ovary from a 100-day-old mouse **(A)**, given sc injections of 10 μl sesame oil:ethanol (9:1; v:v) [vehicle] at 5–7 days of age. Cross-section of ovary from the 100-day-old recipient Tx-3 mouse **(B)**, infused at 15 days of age with 20 million lymphocytes, taken from the vehicle-injected animal. Cross-section of ovary from a 100-day-old mouse **(C)**, given injections of 20 μg E_2 _in 10 μl vehicle at 5–7 days of age. Cross-section of ovary from the 100-day-old recipient Tx-3 mouse **(D)**, infused at 15 days of age with 20 million lymphocytes, taken from the E_2_-injected animal. Cross-section of ovary from a 100-day-old mouse **(E)**, given injections of 10 μg DES in 10 μl vehicle at 5–7 days of age. Cross-section of ovary from the 100-day-old recipient Tx-3 mouse **(F)**, infused at 15 days of age with 20 million lymphocytes, taken from the DES-injected animal.

Study 2 demonstrated the ability of lymphocytes from E_2_-injected mice to create follicular cysts when infused into Tx-3 animals. Female mice, injected at 5–7 days of age with either E_2 _or diethylstilbestrol (DES), were killed at 100–110 days of age, and their lymphocytes (isolated from spleens) infused into 15-day-old, Tx-3 females. Control Tx-3 mice were given lymphocytes from vehicle-injected animals. Recipients were killed at 100 days of age, and ovaries from both donor and recipient mice examined for the presence of follicular cysts and CLs. All ovaries from E_2_-injected (n = 10) and DES-injected (n = 20) donor-animals had follicular cysts; none had CLs. Seven of 10 of recipient mice infused with lymphocytes from E_2_-injected animals, and 11 of 20 infused with lymphocytes from DES-injected animals, had ovaries containing follicular cysts. Taken in toto a reasonable explanation for these results is that the infusate from the E_2_- and DES-injected animals contained autoreactive T cells, but lacked regulatory T cells; a combination that led to the creation of cysts in the recipient Tx-3 mice. The absence of cysts in some Tx-3 recipients is likely due to incomplete thymectomy. Thymic fragments are fully capable of producing regulatory T cells, as we have previously reported [12]. No cysts were seen in the ovaries of Tx-3 mice infused with lymphocytes from vehicle-injected animals. Figure 1 contains photomicrographs of ovaries from: (A) vehicle-injected donor and (B) Tx-3 recipient mice; (C) E_2_-injected donor and (D) Tx-3 recipient mice; and, (E) DES-injected donor and (F) Tx-3 recipient mice. Note CLs in photomicrographs A and B and their absence in photomicrographs C through F.

The remaining studies determined the role of steroids in altering the ability of the thymus to produce regulatory T cells. Normally, thymocytes do not exit the thymus until they become mature T cells. However, estrogen is reported to increase the gland's vascular permeability [13]. This could cause early thymocyte discharge and contravene production of regulatory T cells. Female mice were injected at 5–7 days of age with E_2_, testosterone (T), cortisol, or progesterone. At 12-days of age they were killed, thymuses and spleens removed and weighed, and thymocytes and splenocytes counted. Mice injected with E_2 _had 50 million fewer thymocytes than control animals (p < 0.025) (Table 2). Mice injected with cortisol and progesterone had 30 million and 10 million fewer thymocytes, respectively (N.S.). Notably, animals injected with T had increased numbers of thymocytes. This was unexpected and necessitated a more detailed assessment of the effect of T on the thymus; for comparison, a similar analysis was made of the effect of E_2_.

**Table 2:** The effect of injecting female B6A mice with various steroid hormones at 5–7 days of age on thymus and spleen weights and thymocyte and splenocyte numbers at 12 days of age.

Steroid	Thymus Wt (mg)	Spleen Wt (mg)	Thymocytes (10^6^)	Splenocyes (10^6^)
Vehicle [5] ^a^	50.5 ± 3.2^b^	40.6 ± 3.6	83.0 ± 6.1	33.2 ± 5.9
Progesterone [4]	48.8 ± 3.0	37.4 ± 0.6	73.5 ± 18.1	33.5 ± 1.3
Testosterone [9]	47.8 ± 2.6	46.0 ± 3.6	143.2 ± 25.5	89.2 ± 23.1
Cortisol [13]	26.5 ± 3.4**	27.9 ± 2.0*	52.3 ± 9.2	19.2 ± 3.2
Estradiol-17β [8]	18.5 ± 3.2***	14.6 ± 1.8***	33.6 ± 12.0*	5.8 ± 1.1***

Beginning at 5 days of age, mice were given three daily sc injections of 10 μl sesame oil: ethanol (9:1; v:v) {vehicle}, or vehicle containing 20 μg of the indicated steroid hormones. At 12 days of age they were killed, thymuses and spleens removed and weighed, and thymocytes and splenocytes visualized under a light microscope using the Trypan Blue exclusion test, and counted with a hemocytometer.

^a^Number of animals in each group.

^b^Mean ± S.E.M.

*Significant @ p < .025. **Significant @ p < .005. ***Significant @ p < .001.

**Figure 2:**
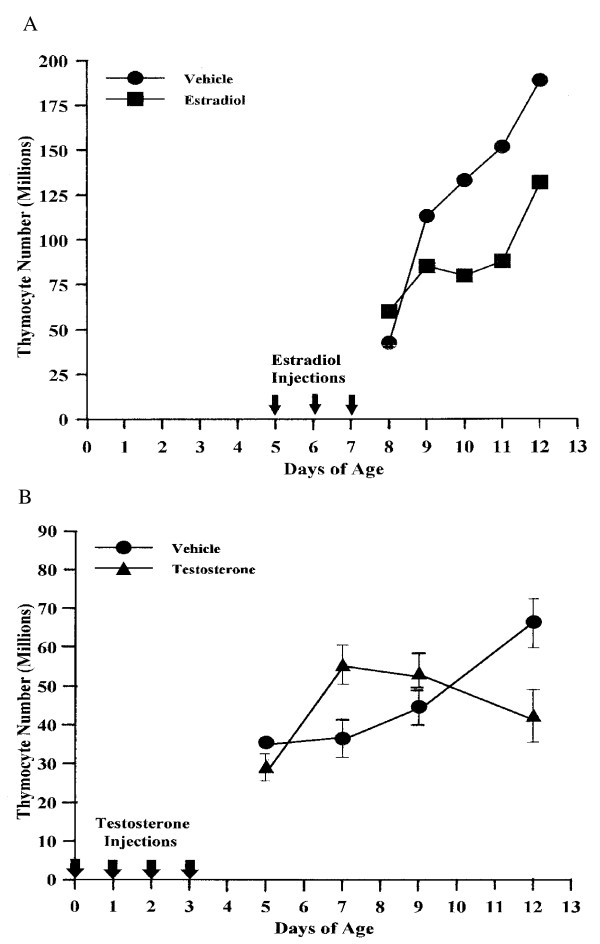
**E_2_- and T-induced changes in thymocyte levels of female B6A mice**. Animals in **(A)** were given 3 daily sc injections of 10 μl sesame oil:ethanol (9:1; v:v) [vehicle] with or without 20 μg E_2_, starting at 5 days of age. They were killed at 8, 9, 10, 11, and 12 days of age. Each data-point consists of the average number of viable thymocytes isolated from thymuses of 2 animals. In **(B)** the animals were given 4 daily injections of either 10 μl vehicle, or vehicle containing 20 μg T, starting at 0 days of age. They were killed at 5, 7, 9, and 12 days of age. Five animals were used for each data-point. Thymocytes were visualized using the Trypan Blue Exclusion Test and counted in a hemocytometer.

Female mice were injected at 5–7 days of age with E_2 _or at 0–3 days of age with T (T having been shown to have a slower time course for producing cysts [5]). Beginning 1 day after the last E_2 _injection and 2 days after T, and at intervals thereafter, the animals were killed, thymuses removed and thymocytes counted. Response to E_2 _was rapid. Beginning two days after the last injection, thymuses of E_2_-injected animals averaged 50 million fewer thymocytes than those of control mice (Fig. 2A). Response to T was quite different (Fig. 2B). Instead of a decrease, thymocyte levels initially increased, reaching a zenith four days after the last injection. At this juncture, thymuses of T-injected mice contained 20 million more thymocytes than those of control animals. Thymocyte levels subsequently fell, and after five days thymuses of T-injected mice contained 27 million fewer thymocytes than those of control mice. The slower rate of thymocyte loss, relative to the rapid loss induced by E_2_, is reflected in the ability of each steroid to cause anovulation and follicular cysts. T is less effective than E_2_, and produces a "delayed anovulation syndrome" [5,14,15].

In adult mice, thymocyte loss after E_2 _administration is unevenly distributed. While the cortex experiences some loss of thymocytes, the greatest decline occurs in the medulla [16]. Prior to E_2_, this region of the thymus contains substantial levels of CD4^+^CD8^+ ^double positive (DP) thymocytes and CD4^+ ^and CD8^+ ^T cells. After E_2_, all three subsets suffer a 99% reduction. Their lowered levels reflect a drastically reduced role for the medulla in T cell development. The loss of thymocytes was interpreted as being due to a blockage in early T cell development [16]. However, a significant influx in thymocytes precedes any loss (Fig. 2B). This response is best explained as resulting from an increase in the thymus' vascular permeability. Data from Abo [17,18] support this interpretation. In his study of extrathymic T cell maturation, Abo noted that sinusoids of mouse liver contain lymphocytes that increase in number after E_2 _injection. Their increase occurs in parallel with thymocyte loss and thymic involution. FACS analysis reveals them as being a mixed population of CD4^-^CD8^- ^double negative (DN) cells with α/β T cell receptor (TcR), and CD4^+ ^and CD8^+ ^T cells [17]. Since DN cells are normally found in the cortex of the thymus, their appearance in the liver suggests a premature exit at, or near, the corticomedullary junction. Further evidence of a truncated pathway is indicated by the observation that estrogen-injected mice have significantly higher splenocyte levels than control animals (Table 3).

**Table 3:** The effect of injecting female B6A mice with estrogen at 5–7 days of age on thymocyte and splenocyte numbers at adulthood.

Age and Treatment	Thymocytes × 10^6^	Splenocytes × 10^6^
60-day-old, vehicle-injected [8]^a^	72.5 ± 12.0^b^	-----
60-day-old, estrogen-injected [14]	43.0 ± 4.8*	-----
100-day-old, vehicle-injected [10]	122.6 ± 19.1	22.0 ± 3.7
100-day-old, estrogen-injected [14]	57.0 ± 7.8**	52.5 ± 5.4*
150-day-old, vehicle-injected [18]	-----	20.4 ± 1.6
150-day-old, estrogen-injected [27]	-----	50.2 + 5.8***

Beginning at 5 days of age, mice were given 3 daily sc injections of 10 μl sesame oil: ethanol (9:1; v:v) {vehicle), with and without 20 μg estradiol-17β. In one study the mice were killed at 60 days of age, thymuses removed, and thymocytes visualized under a light microscope using the Trypan Blue exclusion test and counted in a hemocytometer. In a second study, the mice were killed at 100 days of age, and spleens also removed, and both splenocytes and thymocytes isolated and counted. In a third study, the mice were killed at 150 days of age and only splenocytes were counted.

^a^Number of animals in each group.

^b^Mean ± S.E.M.

*Significant at p < .05. **Significant at p < .01. ***Significant at p < .001.

**Figure 3:**
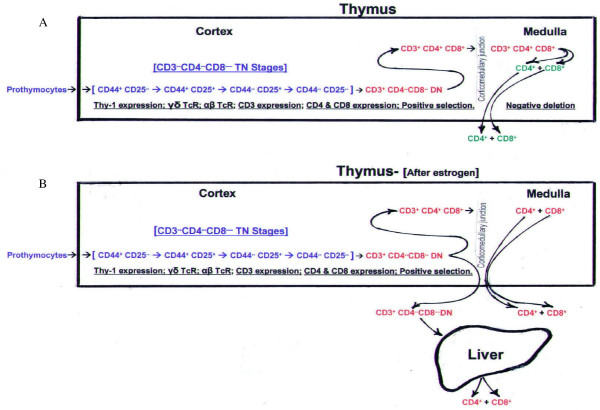
**Proposed pathway for the discharge of immature thymocytes from thymuses of E_2_-injected female B6A mice**. The top diagram **(A)** depicts the typical thymocyte maturation pathway, beginning with the sub-capsular entry of prothymocytes into the cortex and the expression of Thy-1. Maturation continues through CD3^-^CD4^-^CD8^- ^triple negative (TN) stages and the development of the T cell receptor (TcR). Subsequent expression of CD3 produces CD3^+^CD4^- ^CD8^- ^double negative (DN) cells with αβ TcR. After expression of CD4 and CD8, the CD4^+^CD8^+ ^double positive (DP) cells undergo the process of positive selection. Surviving DP cells pass through the corticomedullary junction into the medulla. Here, they are transformed into CD4^+ ^(SP) and CD8^+ ^(SP) T cells, and in the process, autoreactive T cells are eliminated. Mature SP T cells then exit the thymus at the corticomedullary junction. The bottom diagram **(B)** shows how the E_2_-induced increase in vascular permeability is proposed to affect thymocyte maturation. Thymocytes, rather than expressing CD4^+ ^and CD8^+ ^and continuing into the medulla, exit the thymus at the corticomedullary junction. Maturation into SP T cells then takes place in the sinusoids of the liver.

Fig. 3A depicts the normal developmental history of CD4^+ ^and CD8^+ ^T cells. Development begins when bone marrow precursors enter the thymus at the outer, sub-capsular region [19]. As the cells then migrate through the cortex they undergo a number of developmental stages, first as CD3^-^CD4^-^CD8^- ^triple negative (TN) thymocytes, and later, after CD3 expression, as DN thymocytes. During transit the α/β TcR is formed. DN thymocytes express CD4 and CD8 and, as DP thymocytes, interact with cortical epithelial cells to undergo positive selection. Thymocytes survive this process if their TcR, along with CD8 and/or CD4 accessory molecules, selectively binds to MHC class I and class II positive epithelial cells. Surviving DP thymocytes then pass through the corticomedullary junction into the medulla, where they undergo negative deletion. The TcR this time is exposed to self-antigens produced by medullary epithelial cells (MECs). Synthesis of self-antigens, such as tissue proteins, is under the control of the autoimmune regulator (Aire) promoter [20]. It should be noted that binding of the TcR to self-antigens in conjunction with MHC class I and class II molecules causes self-destruction and elimination of most autoreactive T cells. Regulatory T cells control those that are not eliminated and that enter into the system. Regulatory T cells comprise less than 10% of CD4^+ ^T cells, and are identified by their expression of the CD25 antigen (interleukin-2 receptor α-chain) [9]. The CD4^+^CD25^+ ^T cells are unique in that the binding of their TcR to self-antigens results in activation and conversion to a nonproliferative (anergic) state [21,22]. Thus activated they are capable of preventing the activation of autoreactive CD4^+ ^and CD8^+ ^T cells [21]. All mature CD4^+ ^and CD8^+ ^T cells exit the thymus at the corticomedullary junction [19].

Fig. 3B shows the proposed thymocyte maturation pathway after an increase in thymic vascular permeability. Prothymocytes continue to enter at the outer, sub-capsular region, and proceed through stages of TN development, CD3 expression, and formation of the α/β TcR. However, instead of expressing CD4 and CD8, the majority of CD3^+^CD4^-^CD8^- ^(DN) cells exit at the corticomedullary junction, bypassing positive selection and negative deletion. Their subsequent development into T cells has been proposed to occur in the sinusoids of the liver [17,18]. The loss of 50 million thymocytes in E_2_-injected mice (Fig. 2A) is likely due, in large part, to this altered pathway. The long-lasting nature of the new pathway is indicated by the 48 million (average) fewer thymocytes found in 60- and 100-day-old perinatally E_2_-injected mice (Table 3).

The ability of CD4^+^CD25^+ ^regulatory T cells to exert control over CD4^+^_Autoreactive _and CD8^+^_Autoreactive _T cells is attained in a region of the thymus (medulla) that is not fully functional until 7–10 days postpartum [7,19]. Tx-3, therefore, permanently negates development of CD4^+^CD25^+ ^regulatory T cells [9]. E_2 _administration achieves this same result. Instead of physical removal of the thymus, however, E_2 _initiates an altered thymocyte maturation pathway and de facto circumvention of the medulla. The involvement of CD8^+^_Autoreactive _T cells in the formation of follicular cysts is suggested by the report that CD8^+ ^T cells selectively infiltrate ovarian tissue during cyst formation [23]. In a previous study, we made an analysis of the combined effects of Tx-3 and E_2 _on ovarian pathology [12]. Tx-3 by itself was found to have an incidence of ovarian dysgenesis, of 45%. The Tx-3 mice having normal ovaries (55%) were found at autopsy to contain thymic fragments. A second group, in which E_2 _was injected after Tx-3, had a similar efficacy of ovarian dysgenesis (46%). However, instead of the remaining animals having normal ovaries, they either had ovaries that were dysgenic and cystic (39%), or ovaries that were cystic (15%). These results provided a basic understanding of the sequence in T cell development, as is diagrammed below.

CD4^+^_Autoreactive _T cells are the first produced by the thymus. Tx-3 at point A prevents development of CD8^+^_Autoreactive _cells, T_Reg CD4 _cells, and T_Reg CD8 _cells. This results in ovarian dysgenesis. Tx-3 or E_2 _administration at point B forestalls development of T_Reg CD4 _cells and T_Reg CD8 _cells. This causes both ovarian dysgenesis and follicular cysts. E_2 _injection at point C prevents development of T_Reg CD8 _cells, and follicular cysts are produced. Ovarian dysgenesis does not occur because T_Reg CD4 _cells are produced by the medulla prior to E_2 _intervention. Shown in study 1 (Table 1), ovaries of mice in Treatment 5 have the same pathologies as described for the Tx-3 + E_2 _group (dysgenic, dysgenic + cystic, and cystic) [12]; whereas, the ovaries of Treatment 7 animals are normal. This indicates that the infusate from 7-day-old donors, given to Treatment 5 mice lacked T_Reg CD4 _and T_Reg CD8 _cells; whereas, the infusate from adult donors, given to Treatment 7 animals contained T_Reg CD4 _and T_Reg CD8 _cells. These same results occurred in Treatment 4 and Treatment 6 animals, and for the same reason.

An examination of the literature indicates a strong likelihood of thymus involvement in estrogen and/or testosterone-induced anovulation in other animal species. For example, Kincl et al. [24,25] reported that anovulation in E_2_- and T-injected female rats could be prevented by thymocyte infusion. Notably, only thymocytes from adult donors were effective. Thymocytes from 5-day-old animals did not prevent anovulation. In primates the thymus undergoes its final development prenatally [7]. Steroid action would thus occur in utero. This could explain why injections of testosterone propionate (TP) given to pregnant rhesus monkeys on gestational day's 40–55, produces anovulatory female offspring [26,27]. The female offspring have enlarged ovaries with multiple small follicles; an elevated LH/FSH ratio; and, high levels of serum 17αOH-progesterone and testosterone.

Additional evidence of steroid influence in utero is detailed in reports of the consequences of using DES in pregnant women [28-35]. Prescribed from the 1940s until 1971, DES was banned by the FDA due to the large number of reproductive problems in daughters exposed in utero. Problems included an increased rate of primary infertility, oligomenhorrhea, amenorrhea, high levels of androstenedione and testosterone, facial hirsutism, and an elevated LH/FSH ratio. These symptoms are all associated with the formation of cysts [36,37]. Notably, exposure to DES on gestational weeks 9 through 12 produced the highest rate of infertility [35]. This timeframe is coincident with the final developmental stages of the thymus [7].

The identity of the self-antigen(s) that CD8^+^_Autoreactive _T cells regard as nonself, is at present, a matter for conjecture. MECs synthesize approximately 300 ectopic tissue proteins [20]. At least two are involved in autoimmune disease. A peptide epitope of insulin initiates CD8^+^_Autoreactive _T cell destruction of pancreatic β cells [38], and zona pellucida glycoprotein 3 (ZP3) is implicated as the self-antigen involved in ovarian dysgenesis [39]. Synthesized in the ovary by the oocyte and granulosa cells [40], ZP3 is a prime candidate for the self-antigen involved in the formation of follicular cysts. Destruction of granulosa cells by CD8^+^_Autoreactive _T cells would seriously impair the follicle's capacity to synthesize estrogen. Restoration of this ability might explain why injections of FSH cause ovulation in clomiphene-resistant PCOS women without intervention by either exogenous LH or hCG [41].

In conclusion: we have proposed that follicular cysts formed in a popular animal model of PCOS represent an autoimmune disease initiated by steroid administration. An increased incidence of autoimmune disease in DES-exposed women [42], lends further support for the autoimmune nature of PCOS. As maternally derived androgens and estrogens diffusing into the fetal area are limited by the amnion [43], and are normally at nanogram levels, it is unlikely that this source of steroid causes PCOS. The reproductive problems observed with DES came from milligram levels [44]. Potential sources of steroids at this level are phytoestrogens, contained in food supplements and ingested by some pregnant women. The Centers for Disease Control and Prevention, for example, report that 10% of representative samples of women in the United States contain urinary levels in the milligram range, of phytoestrogens found in flax seed [45]. Flax seed and soy bean products cause reproductive problems in female rats [46] and mice [47], and mice suffer thymocyte loss and thymic atrophy when given genistein, the phytoestrogen contained in soy beans [47]. Our future research will determine whether or not phytoestrogens cause anovulation and follicular cysts when administered to female mice during the thymus' critical period. We will also be investigating the impact of adrenal corticoids. While the bulk of this paper has concentrated on the role of gonadal steroids, the observation that adrenal steroids diminish thymic/spleen weight and numbers of thymocytes/splenocytes (Table 2), and can instigate cyst formation [6], raises the possibility that severe stress during pregnancy may be a factor in PCOS development.

## Competing interests

The authors declare that they have no competing interests.

## Authors' contributions

JCC and SDM conceived of the study, participated in its design and coordination, and drafted the manuscript. SHM oversaw the injection of steroids and cell counting, and performed neonatal thymectomy and thymocyte infusion. JCC directed the infusion of lymphocytes. SMF performed the ovarian histology and helped draft the manuscript. All authors read and approved the final manuscript.
